# The immunohistochemical and urodynamic evaluation towards the collagen-coated and non-coated polypropylene meshes implanted in the pelvic wall of the rats

**DOI:** 10.1038/srep38960

**Published:** 2016-12-19

**Authors:** Tsia-Shu Lo, Yi-Hao Lin, Faridah Mohd Yusoff, Hsiao-Chien Chu, Wu-Chiao Hsieh, Ma. Clarissa Uy-Patrimonio

**Affiliations:** 1 Department of Obstetrics and Gynecology, Chang Gung Memorial Hospital, Keelung, Medical Center, Keelung, Taiwan, Republic of China; 2 Division of Urogynecology, Department of Obstetrics and Gynecology, Linkou, Chang Gung Memorial Hospital, Linkou Medical Center, Taoyuan, Taiwan, Republic of China; 3 Chang Gung University, School of Medicine, Taoyuan, Taiwan, Republic of China; 4 Department of Obstetrics and Gynaecology, Hospital Sultanah Nur Zahirah, Kuala Terengganu, Malaysia; 5 Fellow of the Division of Urogynaecology, Department of Obstetrics & Gynaecology, Chang Gung Memorial Hospital, Linkou, Taoyuan, Taiwan, Republic of China; 6 Department of Obstetrics and Gynecology, Dr. Pablo O. Torre Memorial Hospital, Bacolod City, Philippines

## Abstract

Our aim is to study the inflammatory response towards the collagen-coated and non-coated polypropylene meshes in rats and the urodynamic investigation post-operatively. Forty-two female Sprague Dawley were divided into 7 groups of 6 rats; Control, Day 7 and 30 for Sham, Avaulta Plus (MPC), Perigee (MP). UDS were taken at days 7 and 30. Mesh with the vagina and bladder wall was removed and sent for immunohistochemical examination. Results showed intense inflammatory reaction on day 7 in the study groups which decreased on day 30. IL-1, TNF-α, MMP-2 and CD31 were observed to decrease from day 7 to day 30. NGF was almost normal on day 30 in all groups. UDS showed no difference in voiding pressure. Both Study and Sham groups had shorter voiding interval (VI) on day 7 but significantly lower in MPC. VI had significantly increased on day 30 in all groups. Voided volume was significantly lower in the mesh groups even when an increase was seen on day 30. In conclusion, the higher levels of IL-1, TNF-α and MMP-2 in collagen-coated polypropylene mesh imply greater inflammation than the non-coated polypropylene mesh. Mesh implantation can lead to shorter voiding interval and smaller bladder capacity.

Host response towards implanted biomaterials or devices involves a few processes such as injury, inflammatory and wound healing responses, foreign body reactions and ultimately fibrous encapsulation of the materials^1^. The inflammatory process would initiate the release of various chemical mediators. Interleukin-1 (IL-1), Tumour Necrosis Factor-α (TNF-α), Nerve Growth Factor (NGF) Matrix metalloproteinases (MMPs) and angiogenesis (surface antigen CD-31) are among the important inflammatory markers involved. While these markers had been studied in the biocompatibility of the mesh, their effect on the lower urinary tract symptoms (LUTS) are not known except for NGF which had been widely investigated^2^,^3^,^4^.

IL-1 plays a central role in the regulation of immune and inflammatory responses to infections or sterile insults^5^ and is intensely produced by tissue macrophages, monocytes, fibroblasts, and dendritic cells while TNF is mainly secreted by macrophages and can induce cell death of certain tumour cell lines. MMPs are proteolytic enzymes which hydrolyse the compositions of extracellular matrix (ECM) and impact cell movement, growth, differentiation and survival^6^. Among the MMPs, MMP-2 and MMP-9 are the main enzymes responsible for ECM remodelling^7^.

Synthetic mesh may be use for correction of pelvic organ prolapse (POP). However, varieties of LUTS including, de novo overactive bladder^8^,^9^, voiding dysfunction and de novo urinary stress incontinence^10^ may occur after mesh implantation surgery for POP. It would be difficult to differentiate if the LUTS were brought about by a transient physiologic phenomenon secondary to the surgical procedure or a result of a worrisome reaction to the mesh. Once the synthetic mesh is implanted into the vagina, inflammatory reaction will be triggered leading to migration of macrophages and fibroblasts to the implanted site. This reaction may also involve many other cytokines, such as the IL-1, TNF-α, NGF and MMPs.

Our study focused on the inflammatory and immunohistochemical responses of chemical mediators IL-1, TNF-α, NGF, MMP-2 and CD31 towards the polypropylene mesh with and without collagen coating, implanted in the pelvic wall of the rats and their relationship with the functional urodynamic investigations post-operatively. Coated-mesh had been developed so as to improve the success of vaginal prolapse repair^8^ and overcome the graft-related complication such as graft rejection. Studies involving different types of coated-mesh either using dermal or urinary bladder ECM, porcine/bovine collagen had been conducted on different aspects but with the same target as to determine the best and safest mesh implant which is still debatable until now^11^,^12^,^13^.

## Materials and Methods

All experimental protocols and procedures were approved and funded by the Institutional Animal Care and Use Committee of CGMH (No: 2012122504). Procedures done were in accordance with the guidelines and regulations of the same institution.

### Surgical mesh

Two types of mesh were used in this study, Avaulta Plus (C.R. Bard, Inc., Murray Hill, NJ, USA), a porcine collagen-coated macroporous polypropylene mesh (MPC) and Perigee (AMS, Inc., Minnetonka, MN, USA), uncoated macroporous polypropylene mesh (MP).

### Study sample

42 female Sprague Dawley (SD) rats of 12.6 ± 0.9 (range, 12–15) week-old were involved with average weight of 312.1 ± 22.7 (range, 271–341) g. They were divided into 7 groups of 6 rats each; control (group A), sham 7 days (group B), sham 30 days (group C), MPC 7 days (group D), MPC 30 days (group E), MP 7 days (group F) and MP 30 days (group G).

### Surgical procedures

All surgeries were carried out under Isoflurane anaesthesia in an animal laboratory. Cefazolin was served as pre-operative antibiotic prophylaxis. Surgeries were performed by the authors. The rat’s vagina was exposed using a Lonestar retractor. Hydro-dissection was performed by injecting normal saline (0.5–1.0cc) at the anterior vaginal wall followed by a 1 cm midline incision. The space between the vagina and bladder was opened. In MP and MPC groups, a 0.5 × 0.5 cm square mesh was inserted into the opened space and the vaginal mucosa closed with Polyglactin 5/0 suture (Vicryl). The Sham group had similar surgery with no mesh implanted. Buprenex (0.1 mg/kg) was injected subcutaneously for analgesia post-operatively. The rats were sacrificed after CMG analysis. The mesh with the vagina and bladder wall was removed for histological and western blot analysis.

### Suprapubic Tube Implantation (SPT)

Under isoflurane, a midline longitudinal abdominal incision was made, 1 cm above the urethral meatus. The bladder was located, and a circular purse string suture of 5-0 silk was placed on the bladder under stereomicroscope. The PE-50 tubing with a flared tip was implanted, secured by the purse string suture and tunnelled subcutaneously out to the skin. Then the abdominal incision was closed.

### Conscious Cystometrogram (CMG) measurement

Two days after SPT, the rats were placed in special metabolic cages (Med Associates Inc., St. Albans, VT) for CMG for 70–80 minutes. The bladder catheter was connected to the syringe pump and pressure transducer. All bladder pressures were referenced to the air pressure at the level of the bladder. Pressure and force transducer signals were amplified, recorded and digitalised for data collection. The bladder was then filled with 0.9% room-temperature saline at 5 ml/hr through the bladder catheter while the bladder pressure was recorded. Urine weight and other data were recorded as done in the study of Lin *et al*.^14^. Voided volume (VV) was defined as the volume expelled during micturition. Peak voiding pressure (PVP) was measured at the peak of the detrusor contraction. The inter-contraction interval between two successive contractions was calculated in each micturition cycles.

### Immunohistochemical process

The sample was homogenised in lysis buffer (PRO-PREPTM solution, iNtRON BIOTECHNOLOGY). The cell lysis was induced by incubation for 20 minutes on ice. The lysis was centrifuged at 13,000 rpm (4 °C) for 10 minutes, and transfer supernatant to a fresh tube. The protein content of the supernatant was estimated by Bradford method. The sample (30 μg/lane) was mixed with sample buffer containing 10% mercaptoethanol (Sigma). The mixture was heated at 100 °C for 10 minutes and applied to a 10% sodium dodecyl sulfate polyacrylamide gel for electrophoresis. The protein was electrophoretically transferred onto nylon membranes. Nonspecific binding was blocked 1 hour at room temperature with 10% (w/v) milk. After washing the membranes with TBS containing 0.1% (v/v) Tween 20 for 3 times, for 10 minutes each, were incubated overnight at 4 °C with the antibody 1:1000 dilution. After rinsing in the TBST for 3 times, for 10 minutes each, were incubated with goat anti-rabbit IgG horseradish peroxidase conjugate antibody (SIG-A0545, sigma, 1:10000). The membranes were incubated in the chemiluminescence reagent as described by Lin *et al*.^14^.

### Outcome measures

The outcomes measured were the density of inflammatory reaction produced by the IL-1, TNF-α, NGF, MMP-2 and CD-31 around the surgical site/area of implantation and their association with the functional urodynamic investigation of the SD rats.

### Statistical analysis

Descriptive statistics were used in analysis of NGF, IL-1, TNF-α, MMP-2 and CD-31 results. ANOVA and Fisher exact test were applied for comparison. Values of p < 0.05 were considered statistically significant. All statistical methods were performed using the commercial software SPSS, version 17.

## Results

**Figure 1:**
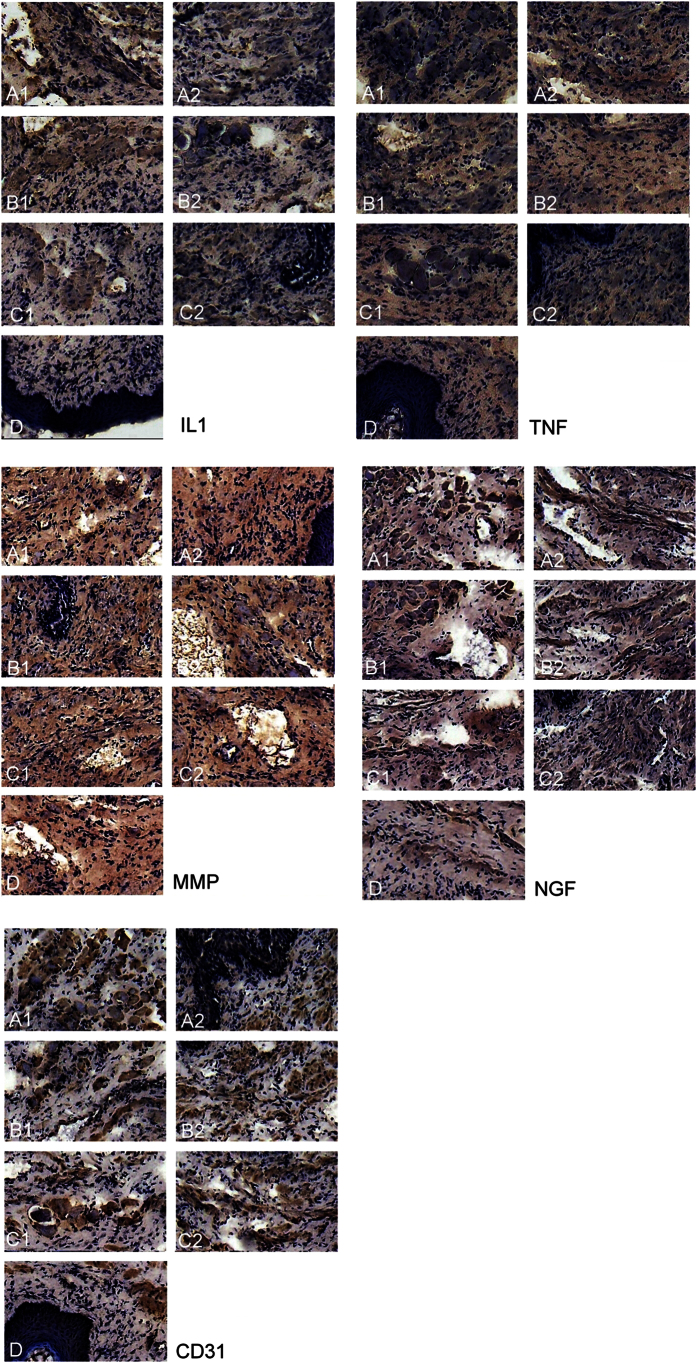
Analysis of immunoreactive expression of IL-1, TNF-α, MMP-2, NGF and CD31at 7 days and 30 days post-mesh implant. A1, 7 days post implant on MP group; A2, 30 days post implant on MP group; B1, 7 days post implant on MPC group; B2, 30 days post implant; on MPC group C1, 7 days post implant on sham group; C2, 30 days post implant on sham group; D, control group. *Brown spots signifies inflammatory cells (Reagents: anti-NGF/TA300799/origene; anti-IL1 antibody/TA336742/origene; anti-MMP2 antibody/TA336592/origene; anti-TNF antibody/PA5-19810/Thermo; anti-CD31 antibody/PA5-24411/Thermo).

**Figure 2:**
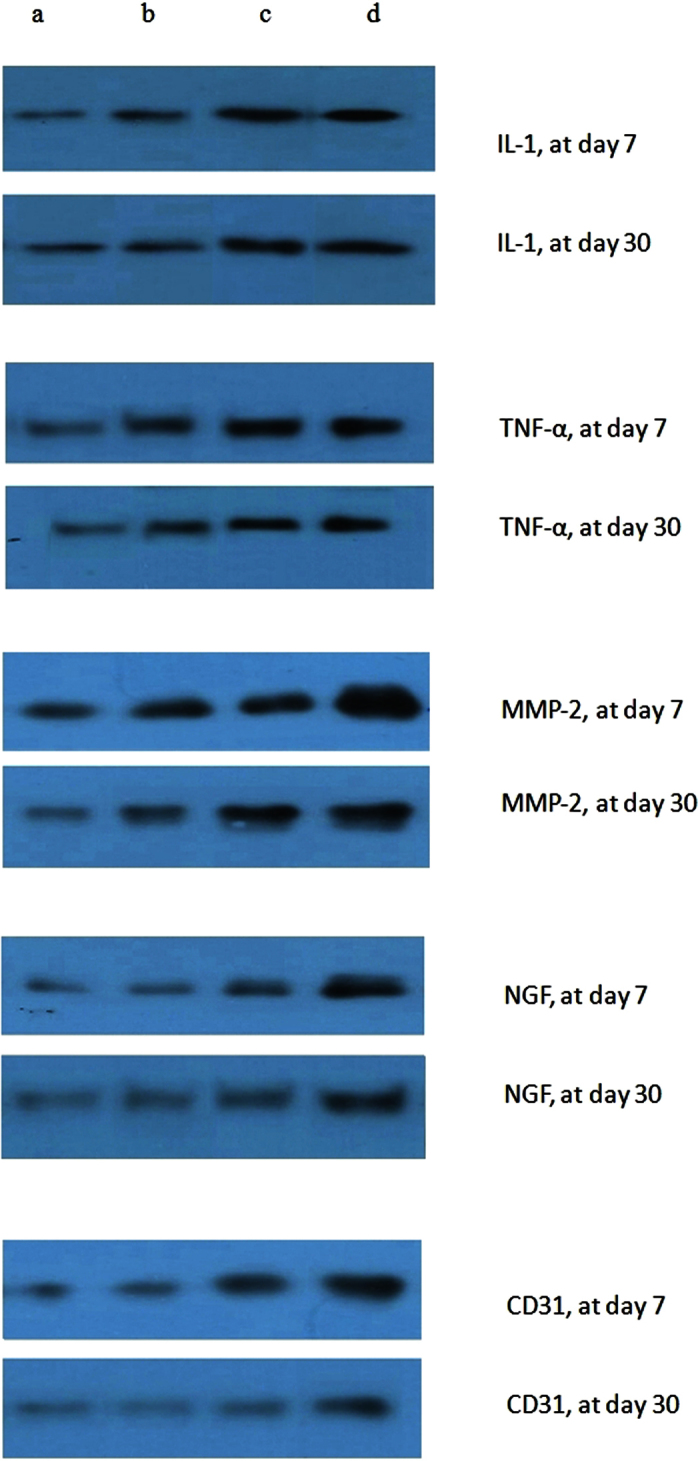
Western blot analysis in IL-1, TNF-α, MMP-2, NGF and CD31. The figure shows increased expression in the sham, MP, and MPC compared with control group at day 7, and in the sham, MP, and MPC compared with control group at day 30 (**a**) Control. (**b**) Sham group. (**c**) MP group. (**d**) MPC group.

**Figure 3:**
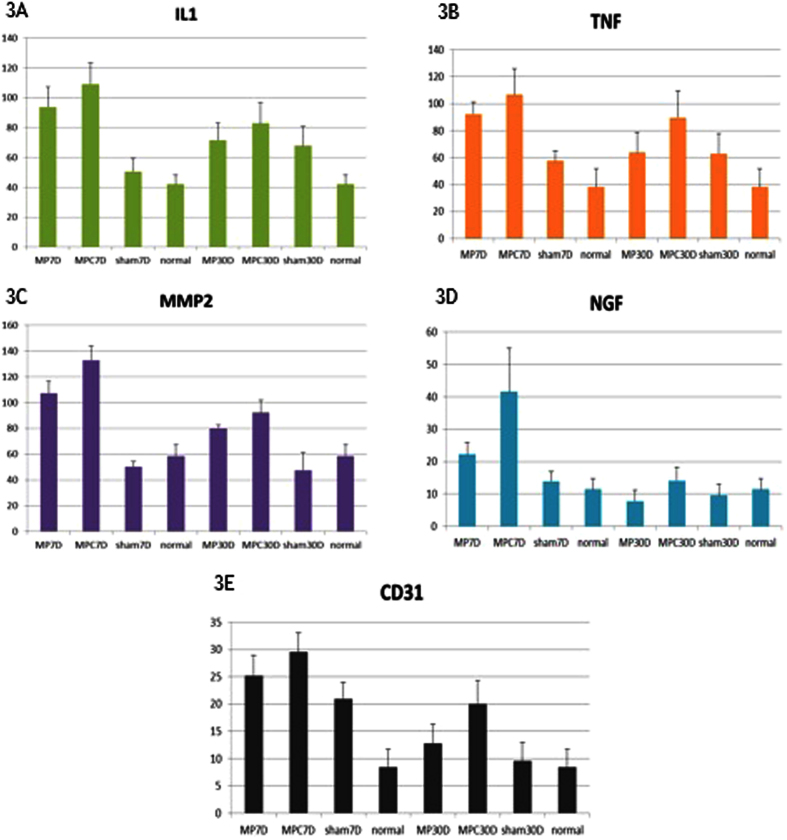
The magnitude of changes in immunohistochemical evaluation of IL-1 (3A), TNF-α (3B), MMP-2 (3C), NGF(3D) and CD31(3E) on Day 7 and Day 30 after transvaginal mesh surgery in SD rats. Legend: MP- uncoated polypropylene mesh, MPC- collagen-coated polypropylene mesh, Sham – no mesh implanted, Normal – control, 7D – day 7, 30D – day 30.

All rats survived and no complications were observed during the post implant period. No dehiscence or mesh exposure was seen at the implant sites. Table 1 showed the immunohistochemical analysis of IL-1, TNF-α, MMP-2, NGF and CD-31. The reaction was significantly more intense in the mesh group than the sham and normal groups, where MPC showed a larger area of inflammation as compared to MP with p < 0.001. TNF-α, MMP-2 and CD-31 were observed to decrease from day 7 to day 30 in MP and MPC groups but despite the decline, the level in the MPC group was still significantly higher than the MP group. IL-1 showed similar changes but there was no significant difference between MP and MPC. There was no significant change seen in the sham and normal groups. The NGF returned towards normal level on day 30 in all groups (Fig. 1). Western blot analysis on IL-1, TNF-α, MMP-2, NGF and CD-31 study have shown increased expression in the MPC, MP, over sham and control (Fig. 2). The intensity of IL-1, TNF-α, MMP-2, NGF and CD-31 was decreased at day 30 when compared day 7 (Fig. 3).

**Figure 4:**
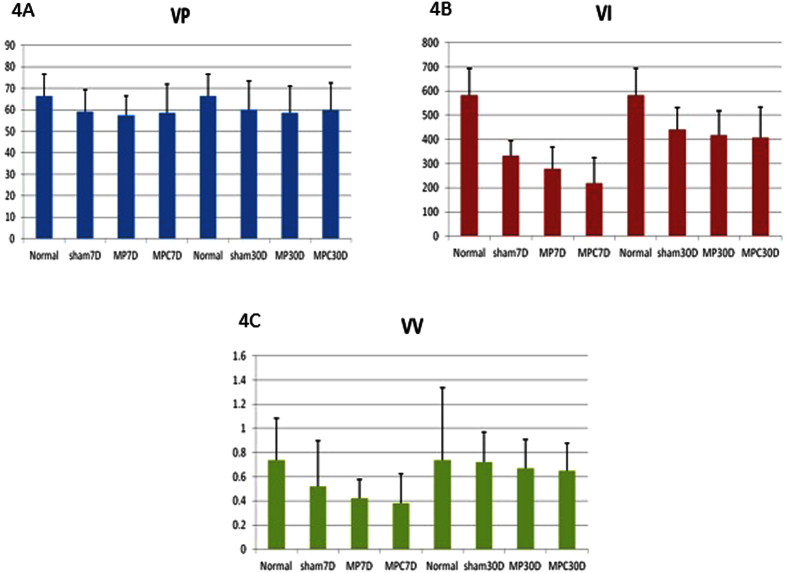
The magnitude of changes in post-operative Urodynamic study, VV (4A), VI (4B), VV(4C). Legend: VP - Voiding pressure, VI – Voiding Interval, VV – Voiding Volume, MP - uncoated polypropylene mesh, MPC - collagen-coated polypropylene mesh, Sham – no mesh implanted, Normal – control, 7D – day 7, 30D – day 30.

The UDS parameters among the groups were shown in Table 2. There was no difference in the voiding pressure (VP) between the groups on days 7 and 30. For the voiding interval (VI), both the Study and Sham groups had a shorter VI when compared to the normal group on day 7 and MPC had significantly lower VI when compared to MP. The VI had significantly increased on day 30 in all groups. Meanwhile, the VV was significantly lower in the mesh groups as compared to the sham even when an increase was seen on day 30 post-operation (Fig. 4).

## Discussion

Clinically, the collagen-coating was claimed to serve as a barrier against mesh exposure^15^. However, the addition of collagen-coating has an impact on persistent high chemical mediators’ immunoreactivity as observed in our study. This suggests that the presence of synthetic mesh as a foreign body may prolong the inflammatory phase especially with the collagen-coated mesh. Ultrasound features have proven that degeneration of the collagen barrier may take longer than expected, and integration of collagen-coated mesh could occur up to 1 year^8^. So, even if the collagen-coated mesh was less integrated in the vaginal wall^16^, the inflammatory reaction was prolonged which allows one to propose that adverse events such as delayed infection, mesh erosion and even the LUTS observed in clinical setting might depend on the duration of the integration process in the collagen-coated mesh. However, Dias *et al*. reported a decrease in the average density of MMP-2 expression in the collagen-coated polypropylene mesh after 90 days and there was no significant difference in the immunological response (IL-1) and tissue necrosis/apoptosis (TNF-α)^17^ in contrast with our findings. This aspect of collagen-coated mesh needs to be looked into as the effect of prolonged inflammatory reaction in this type of mesh is still not clear.

The use of lightweight, macroporous monofilament polypropylene mesh had been popular in many years among the urogynecologist. These properties of the mesh maintain strength, increase flexibility, and decrease load on the tissues^9^. It also reduced the immunogenic response and offered the greatest biocompatibility^18^. Avaulta Plus used in our study is a type of such mesh but coated with porcine collagen. Inflammatory response to macroporous monofilament polypropylene mesh had been described in some studies^8^,^19^,^20^. Elmer *et al*. found that this mesh induced a mild, persistent foreign body reaction with no significant change in the cell count which primarily involved in the inflammatory process^20^. A stiffer, less porous mesh may cause the vagina to produce maladaptive remodelling responses with reduced collagen and elastin content^21^.

The chemistry of the biomaterial surface is important in determining its susceptibility to biodegradation^22^. The incorporation of collagen into the polypropylene mesh is meant to modulate the inflammatory response which may impact the biocompatibility and function of the mesh but Feola *et al*. found that it did not reduce the number of graft-related complications, in fact, the number of infection in Avaulta Plus group was higher^11^. Even though there were more signs of infection, the collagen-coated mesh was less integrated into the vaginal wall with the advantage of reduced tissue adhesion and hence vaginal erosion^15^. Our study showed intense inflammatory reaction in the mesh groups involving IL-1, TNF-α, MMP-2, CD-31 and NGF in early days after implantation consistent with inflammatory response towards injury following a mesh implantation. This reaction was markedly increased on day 7 and declined afterwards in MP and MPC as compared to sham and control groups. Yildirim *et al*. also observed marked inflammation on the early days after implantation and predominant fibrotic reaction on days 15 and 30^23^. Similarly, another study reported an early increase in IL-1 with peak during first week and reduced after 15 days with a sharp increase in MMP-2 around day 7 and declines thereafter^24^.

Chemokines control migration and activation of inflammatory cells. IL-1 and TNF-α, are potent pro-inflammatory cytokines regulated by MMPs. They also play a role in recruitment and activation of inflammatory cells^1^. This may explain the increased recruitment and activation of inflammatory cells with the magnitude being more in the mesh groups, especially in the collagen-coated mesh. The heterogeneity of TNF level indicates that in clinical practice, patients underwent surgical mesh procedure under apparently similar conditions would result in different mesh conditions. Meanwhile, NGF is a small secreted protein produced by the urothelium and smooth muscle which induces the differentiation and survival of particular target neurons^25^. We have observed that transvaginal mesh implantation in the SD rats was associated with significant increase in expression of NGF in the first week which returned to the almost normal level after 30 days. This indicates an active involvement of NGF in acute inflammatory reaction and for the past years, the role of NGF as the biomarkers in overactive bladder, interstitial cystitis and other LUTS had been actively investigated and reviewed^2^,^3^,^4^,^26^,^27^. CD-31 was also increased in early days post-implantation which signifies a normal reaction to inflammation which is angiogenesis.

In our study, the mesh groups present a significant magnitude of reduction in MMP-2 immunoreactivity than sham on day 30 which may indicate stabilization of the extracellular remodelling. However, MPC has shown a higher expression of MMP-2 than MP which is a sign in favour of biocompatibility of the non-coated mesh. MMP is a production of fibroblast and an indicator of early inflammatory activity. It is important in tissue remodelling process and may show a permanent reaction when a permanent implant is present^25^. Liang *et al*. observed an increase in active MMPs including MMP-2 at 1 year after mesh implantation which means active remodelling persists after a long period of time^28^. A recent study showed that fibroblasts surrounding the mesh displayed a strong MMP-2 gene transcriptions whereas farther fibroblast had low MMP-2 synthesis rate^29^.

Urodynamic parameters showed no difference in the VP among the groups but there was a significant increase in frequency and decrease in the VV especially in the early days post-operation. VV remained significantly lower on day 30 in the mesh groups as compared to sham. This observation coincides with the on-going acute and chronic inflammatory events on days 7 and 30 respectively, except for NGF which returned to almost normal level on day 30, the other inflammatory markers surrounding the mesh were significantly present in higher density as compared to the sham group.

In conclusion, the higher levels of IL-1, TNF-α, CD-31 and MMP-2 in the collagen-coated polypropylene mesh than the non-coated polypropylene mesh imply that the presence of collagen-coating triggers greater inflammation. The mesh implantation leads to a shorter voiding interval and smaller bladder capacity. The involvement of IL-1, TNF-α and MMP-2 in the development of de novo LUTS post vaginal mesh implantation has not been explored before. Future studies are needed to find the association of increased inflammatory markers to the development of lower urinary tract symptoms post-mesh implantation surgeries.

## Additional Information

**How to cite this article**: Lo, T.-S. *et al*. The immunohistochemical and urodynamic evaluation towards the collagen-coated and non-coated polypropylene meshes implanted in the pelvic wall of the rats. *Sci. Rep.* **6**, 38960; doi: 10.1038/srep38960 (2016).

**Publisher's note:** Springer Nature remains neutral with regard to jurisdictional claims in published maps and institutional affiliations.

## Figures and Tables

**Table 1:** Immunohistochemistry analysis of IL-1, TNF-α, MMP-2 and NGF after transvaginal mesh surgery in SD rats.

IL-1		Density average	p value	p value*	p value**
			(within group)	(between group)	(between group)
7 days	Normal	42.2 ± 6.3			
n = 6	Sham	67.9 ± 13.0			
n = 6	MP	93.7 ± 13.4		<0.001	
n = 6	MPC	109.2 ± 14.2		<0.001	0.114
30 days	Sham	50.6 ± 8.8	0.089		
n = 6	MP	61.8 ± 11.4	0.040	0.627	
n = 6	MPC	82.9 ± 14.0	0.020	0.017	0.205
TNF-α					
7 days	Normal	38.2 ± 13.7			
n = 6	Sham	63.2 ± 14.4			
n = 6	MP	92.2 ± 9.2		<0.001	
n = 6	MPC	106.7 ± 19.2		0.001	0.165
30 days	Sham	57.6 ± 7.5	0.393		
n = 6	MP	63.4 ± 14.8	0.023	0.955	
n = 6	MPC	89.7 ± 20.0	0.047	0.043	0.048
MMP-2					
7 days	Normal	58.6 ± 8.9			
n = 6	Sham	50.2 ± 4.3			
n = 6	MP	107.4 ± 9.0		<0.001	
n = 6	MPC	132.8 ± 11.3		<0.001	<0.004
30 days	Sham	47.6 ± 13.8	0.758		
n = 6	MP	80.2 ± 2.6	0.006	0.001	
n = 6	MPC	92.2 ± 9.7	0.010	<0.001	0.028
NGF					
7 days	Normal	11.4 ± 3.4			
n = 6	Sham	14.0 ± 3.0			
n = 6	MP	22.2 ± 3.7		0.005	
n = 6	MPC	41.5 ± 13.6		0.002	0.015
30 days	Sham	9.6 ± 3.4	0.151		
n = 6	MP	7.9 ± 3.4	0.005	0.453	
n = 6	MPC	14.1 ± 4.2	0.007	0.102	0.034
CD31					
7 days	Normal	8.4 ± 3.4			
n = 6	Sham	21.0 ± 3.0			
n = 6	MP	25.2 ± 3.7		0.012	
n = 6	MPC	29.5 ± 3.6		0.001	0.011
30 days	Sham	9.6 ± 3.4	<0.001		
n = 6	MP	12.9 ± 3.4	<0.001	0.031	
n = 6	MPC	20.1 ± 4.2	0.001	<0.001	<0.001

*Mesh and Sham; **Mesh with and without collagen-coated.

**Table 2:** Post-operative Urodynamic study (UDS) parameters in normal, sham, non-coated (MP) and collagen-coated polypropylene mesh (MPC) groups.

VP (cmH_2_O)			p value	p value*	p value**
			(within group)	(between group)	(between group)
7 days	Normal	66.3 ± 10.2			
n = 6	Sham	59.1 ± 10.3			
n = 6	MP	57.5 ± 8.8		0.226	
n = 6	MPC	58.5 ± 13.5		0.386	0.564
30 days					
n = 6	Sham	60.1 ± 13.3	0.449		
n = 6	MP	58.5 ± 12.6	0.394	0.342	
n = 6	MPC	59.6 ± 12.9	0.284	0.582	0.341
VI (sec)					
7 days	Normal	583.3 ± 412.3			
n = 6	Sham	331.3 ± 61.6			
n = 6	MP	277.8 ± 200.8		0.010	
n = 6	MPC	218.7 ± 106.7		<0.001	0.031
30 days					
n = 6	Sham	441.5 ± 100.0	<0.001		
n = 6	MP	417.7 ± 100.3	<0.001	0.128	
n = 6	MPC	406.8 ± 125.7	<0.001	0.087	0.258
VV(μl)					
7 days	Normal	0.73 ± 0.34			
n = 6	Sham	0.51 ± 0.27			
n = 6	MP	0.42 ± 0.17		0.015	
n = 6	MPC	0.38 ± 0.24		<0.001	0.115
30 days	Sham	0.71 ± 0.25	<0.001		
n = 6	MP	0.67 ± 0.23	<0.001	0.453	
n = 6	MPC	0.64 ± 0.22	<0.001	0.102	0.234

*Mesh and Sham; **Mesh with and without collagen-coated VP, voiding pressure; VV, voiding volume; VI, voiding interval. Data listed as mean ± standard deviation with 95% confidence intervals in parentheses.

## References

[b1] Anderson J. M. Biological responses to materials. Ann Rev. Mater. Res. 31, 81–110 (2001).

[b2] Lowe E. M. . Increased nerve growth factor levels in the urinary bladder of women with idiopathic sensory urgency and interstitial cystitis. Br J Urol. 79, 572–7 (1997). 9126085 10.1046/j.1464-410x.1997.00097.x

[b3] Vijaya G. . Reliability and validity of urinary nerve growth factor measurement in women with lower urinary tract symptoms. Neurourol Urodyn, doi: 10.1002/nau.22832 (2015). 26227147

[b4] Liu H. T., Tyagi P., Chancellor M. B. & Kuo H. C. Urinary nerve growth factor but not prostaglandin E2 increases in patients with interstitial cystitis/bladder pain syndrome and detrusor overactivity. Br J Urol. 106, 1681–1685 (2009). 10.1111/j.1464-410X.2009.08851.x 19751258

[b5] Ito Y. . IL-1 as a target in inflammation. Endocr Metab Immune Disord Drug Targets. 15(3), 206–11 (2015). 26333726

[b6] Sternlicht M. D. & Werb Z. How matrix metalloproteinases regulate cell behaviour. Annu. Rev. Cell Dev. Biol 17, 463–516 (2001). 11687497 10.1146/annurev.cellbio.17.1.463 PMC2792593

[b7] Page-McCaw A., Ewald A. J. & Werb Z. Matrix metalloproteinases and the regulation of tissue remodelling. Nat Rev Mol Cell Biol 8, 221–233 (2007). 17318226 10.1038/nrm2125 PMC2760082

[b8] Lo T. S. . Assessment of collagen versus non collagen coated anterior vaginal mesh in pelvic reconstructive surgery: prospective study. Eur J Obstet Gynecol Reprod Biol. 198, 138–44 (2016). 26849040 10.1016/j.ejogrb.2016.01.004

[b9] Lo T. S., Tan Y. L., Khanuengkitkong S. & Dass A. K. Surgical outcomes of anterior trans-obturator mesh and vaginal sacrospinous ligament fixation for severe pelvic organ prolapse in overweight and obese Asian women. Int Urogynecol J 24, 809–816 (2013). 23093321 10.1007/s00192-012-1940-7

[b10] Lo T. S., Karim N. B., Nawawi E. A., Wu P. Y. & Nusee Z. Predictors for de novo stress urinary incontinence following extensive pelvic reconstructive surgery. Int Urogynecol J. 26(9), 1313–9 (2015). 25862240 10.1007/s00192-015-2685-x

[b11] Wolf M. T. . Macrophage polarization in response to ECM coated polypropylene mesh. Biomaterials 35, 6838–6849 (2014). 24856104 10.1016/j.biomaterials.2014.04.115 PMC4347831

[b12] Hilger W. S. . Histological and Biomechanical evaluation of implanted graft in a rabbit vaginal and abdominal model. Am J Obstet Gynecol 195, 1826–31 (2006). 17026951 10.1016/j.ajog.2006.07.006

[b13] Feola A. . Host reaction to vaginally inserted collagen containing polypropylene implants in sheep. Am J Obstet Gynecol 212, 474 e1-8 (2015). 10.1016/j.ajog.2014.11.008 25446700

[b14] Lin Y. H. . Effect of suburethral prolene mesh for suburethral function and histology in a stress urinary incontinence mouse model. Int J Urol. 22(11), 1068–74 (2015). 26332139 10.1111/iju.12888

[b15] Schönleben F., Reck T., Tannapfel A., Hohenberger W. & Schneider I. Collagen foil (TissuFoil E) reduces the formation of adhesions when using polypropylene mesh for the repair of experimental abdominal wall defects. Int J Colorectal Dis 21, 840–846 (2006). 16520931 10.1007/s00384-006-0091-z

[b16] de Tayrac R., Alves A. & Thérin M. Collagen-coated vs non-coated low-weight polypropylene meshes in a sheep model for vaginal surgery. A pilot study. Int Urogynecol J 18, 513–520 (2007). 10.1007/s00192-006-0176-9 16941070

[b17] Dias F. G., Prudente A., Siniscalchi R. T., de Vidal B. C. & Riccetto C. L. Can highlty purified collagen coating modulate polypropylene mesh immune-inflammatory and fibroblastic reaction? Immunohistochemical analysis in a rat model. Int. Urogynecol J 26, 569–576 (2015). 25335748 10.1007/s00192-014-2529-0

[b18] Orenstein S. B., Saberski E. R., Kreutzer D. L. & Novitsky Y. W. Comparative analysis of histopathologic effects of synthetic meshes based on material, weight and pore size in mice. J Surg Res. 176, 423–429 (2012). 22099590 10.1016/j.jss.2011.09.031

[b19] Pierce L. M., Asarias J. R., Nguyen P. T., Mings J. R. & Gehrich A. P. Inflammatory cytokine and matrix metalloproteinase expression induced by collagen-coated and uncoated polypropylene meshes in a rat model. Am J Obstet Gynecol 205, 82.e1-9 (2011). 10.1016/j.ajog.2011.02.045 21497787

[b20] Elmer C., Blomgren B., Falconer C., Zhang A. & Altman D. Histological inflammatory response to transvaginal polypropylene mesh for pelvic reconstructive surgery. J Urol. 181, 1189–95 (2009). 19152931 10.1016/j.juro.2008.11.030

[b21] Liang R. . Vaginal degeneration following implantation of mesh with increased stiffness. BJOG 120, 233–243 (2012). 10.1111/1471-0528.12085 PMC3531826 23240802

[b22] Anderson J. M., Rodriguez A. & Chang D. T. Foreign body reaction to biomaterials. Semin Immunol. 20, 86–100 (2008). 18162407 10.1016/j.smim.2007.11.004 PMC2327202

[b23] Yildirim A. . Tissue reactions of 5 sling materials and tissue material detachment strength of a 4 synthetic mesh materials in a rabbit model. J Urol. 174, 2037–40 (2005). 16217389 10.1097/01.ju.0000176478.17749.07

[b24] Souza-Pinto F. J. . Inducible nitric oxide synthase inhibition increases MMP-2 activity leading to imbalance between extracellular matrix deposition and degradation after polypropylene mesh implant. J Biomed Mater Res A. 101, 1379–87 (2013). 23077110 10.1002/jbm.a.34440

[b25] Steers W. D., Kolbeck S., Creedon D. & Tuttle J. B. Nerve growth factor in the urinary bladder of the adult regulates neuronal form and function. J Clin Invest 88, 1709–15 (1991). 1939656 10.1172/JCI115488 PMC295710

[b26] Kuo H. C. Potential Biomarkers Utilized to Define and Manage Overactive Bladder Syndrome. Low Urin Tract Symptoms. 4, 32–41 (2012). 26676698 10.1111/j.1757-5672.2011.00131.x

[b27] Seth J. H. . Nerve growth factor (NGF): a potential urinary biomarker for overactive bladder syndrome (OAB)? BJU Int. 111, 372–380 (2013). 23444927 10.1111/j.1464-410X.2012.11672.x

[b28] Liang R., Zong W., Palcsey S., Abramowitch S. & Moalli P. A. Impact of prolapse meshes on the metabolism of vaginal extracellular matrix in rhesus macaque. Am J Obstet Gynecol 212, 174 e1-7 (2015). 10.1016/j.ajog.2014.08.008 PMC4312539 25128444

[b29] Wu M. P., Huang K. H., Long C. Y., Yang C. C. & Tong Y. C. *In vitro* extracellular matrix model to evaluate stroma cell response to transvaginal mesh. Neurourol Urodyn. 33, 449–54 (2014). 23775843 10.1002/nau.22425

